# Instantaneous wave-free ratio and fractional flow reserve in clinical practice

**DOI:** 10.1007/s12471-018-1125-1

**Published:** 2018-06-19

**Authors:** R. Pisters, M. Ilhan, L. F. Veenstra, B. C. G. Gho, M. Stein, J. C. A. Hoorntje, S. Rasoul

**Affiliations:** 1 Department of Cardiology, Zuyderland Medical Centre, Heerlen, The Netherlands; 2 Department of Cardiology, Maastricht University Medical Centre, Maastricht, The Netherlands; 3 Department of Cardiology, Rijnstate, Arnhem, The Netherlands

**Keywords:** Coronary stenosis, Fractional flow reserve, iFR

## Abstract

**Objectives:**

To compare fractional flow reserve (FFR) and instantaneous wave-free ratio (iFR) measurements in an all-comer patient population with moderate coronary artery stenoses.

**Background:**

Visual assessment of the severity of coronary artery stenoses is often discordant in moderate lesions. FFR allows reliable functional severity assessment in these cases but requires adenosine-induced hyperaemia with associated additional time, costs and side effects. The iFR is a hyperaemia-independent index.

**Methods and results:**

Between November 2015 and February 2017, 356 consecutive patients were included in whom 515 coronary stenoses were measured using both iFR and FFR. Mean iFR and FFR were 0.90 ± 0.09 and 0.86 ± 0.08, respectively. iFR correlated well with FFR [*r* = 0.75; *p* < 0.001]. Receiver operating characteristic analysis identified an area under the curve of 0.92. An iFR-only strategy with a treatment cut-off ≤0.89 revealed a diagnostic classification agreement with the FFR-only strategy in 420 lesions (82%) with a sensitivity of 87%, a specificity of 80%, a positive predictive value of 56% and a negative predictive value of 96%.

**Conclusions:**

Real-time iFR measurements have good negative predictive value compared to FFR, but moderate diagnostic accuracy (82%). It exposes fewer patients to adenosine, reduces procedure time and costs. Further prospective trials are needed to evaluate specific clinical settings, cut-off values and endpoints.

## Take home message (clinical perspective)

- What is known?
  Functional flow reserve (FFR) outperforms visual assessment in moderate coronary artery stenosis but remains underused.
- What is new?
  Although instantaneous wave-free ratio (iFR) has an excellent diagnostic performance FFR discordant measurements occur.
- What is next?
  Evaluation of iFR-FFR mismatches.

## Introduction

Visual and functional assessments of the severity of coronary artery stenoses are often discordant in moderate lesions [1]. Fractional flow reserve (FFR) allows a reliable functional severity assessment in these cases and has become the gold standard. However, it does require adenosine-induced maximal hyperaemia [2] with associated additional side effects, time and costs.

The instantaneous wave-free ratio (iFR) is a new, adenosine-independent index of coronary artery stenosis severity. Whereas the used pressure ratio (that is, distal transstenotic to proximal aortic pressure) does not differ between iFR and FFR, its recording timing does. As opposed to the several full cardiac cycles averaged in FFR, the iFR is recorded during the most stable and minimised coronary resistance: a specific diastolic wave-free period in several cardiac cycles [3]. iFR shows a good classification agreement with FFR [4] and promising results from a hybrid iFR-FFR approach [5]. Two recent large randomised clinical trials demonstrated non-inferiority of an iFR compared to FFR guided revascularisation strategy regarding major adverse cardiovascular events [6, 7]. However, real-life data on concomitant iFR-FFR measurements are scarce but could be useful to improve our understanding of discrepancies, cut-off values and consequently coronary revascularisation outcome [8–13]. We therefore aimed to prospectively compare real-time FFR and iFR measurements in patients with moderate coronary stenosis.

## Methods

### Population

A prospective registry at Zuyderland Medical Centre of all-comers between November 2015 and February 2017 in which intermediate coronary stenoses (i. e. 50–90% diameter stenosis by visual assessment) were measured using both iFR and FFR.

### Procedural aspects

Coronary angiogram was acquired via either a radial (preferred) or femoral approach with administration of, 50 U/kg unfractionated heparin. When a radial approach was used an intra-arterial vasodilator cocktail (nitroglycerin 100 mcg and verapamil 2.5 mg) was administered. A 0.014-inch pressure sensor-tipped wire (PrimeWire Prestige, Volcano Corporation, San Diego, USA) was positioned at the tip of a guiding catheter. After pressure equalisation at the tip of the catheter, the wire was advanced into the target vessel as distally as reasonably possible for pressure recordings. First, iFR was automatically calculated online using the Volcano CORE System version 3.3.0 (Volcano Corporation). Subsequently, FFR was measured during adenosine-induced hyperaemia either via central intravenous administration [at 140 μg/kg/min] or an intracoronary bolus 100–150 μg. At the end of each measurement, the pressure sensor was retracted to the catheter tip to preclude pressure drift. Use of intracoronary nitroglycerin injection was left at the discretion of the cardiologist. Clinical decisions were based exclusively on the currently recommended FFR treatment cut-off value of ≤0.8 because at that time the results from the DEFINE-FLAIR [6] and iFR-SWEDEHEART [7] trials were unknown.

### Diagnostic strategies

The gold standard consisted of an FFR-only approach using a cut-off value of 0.8 to defer or treat when the measurement was higher or lower, respectively. For the iFR-only strategy, we used a cut-off value of ≤0.89 based on limited available data [13]. Finally, we tested the proposed hybrid iFR-FFR approach [13] incorporating an ‘iFR grey zone’ to revascularise (iFR <0.86), defer percutaneous coronary intervention (>0.93) or to require a subsequent FFR measurement to decide (iFR 0.86–0.93).

### Statistical analysis

We used SPSS statistical software version 22.0 (SPSS Inc., Chicago, Illinois) to perform data analysis. Continuous variables are reported as mean (SD) or median (25^th^–75^th^ percentiles) and categorical variables as number of observed patients (percentage). We used Fisher’s exact test when we compared categorical variables between groups and the Student’s t test when we compared normally distributed continuous variables between two groups. If the continuous variable did not follow a normal distribution, we used the Mann-Whitney U test when we drew a comparison between two groups. Correlation between FFR and mean iFR was assessed with Spearman’s rank correlation coefficient (rs). Conventional summary statistics for diagnostic tests, compared with a patient’s true disease status as indicated by FFR ≤0.80, were calculated from a 2 × 2 contingency table, comparing either the iFR-only strategy or the hybrid iFR–FFR strategy with standard FFR. The area under the receiver operating characteristic (ROC) curve was assessed through nonparametric ROC analysis. Subsequently, the optimal mean iFR threshold was verified using the minimally important change (MIC) threshold as the cut-off level, corresponding to a 45-degree tangent line intersection.

### Ethics

The investigation conforms with the principles outlined in the Declaration of Helsinki.

## Results

Between November 2015 and February 2017, a total of 356 consecutive, predominantly (69%) male patients, aged 67 ± 10 years were enrolled, in whom 515 intermediate coronary stenoses were measured using both iFR and FFR. All clinical decisions were based on the FFR measurement using central intravenous adenosine administration in 45% and an intracoronary bolus in the remainder of patients. FFR and iFR measurements were technically simple and feasible in all patients, without procedure-related complications.

**Fig. 1:**
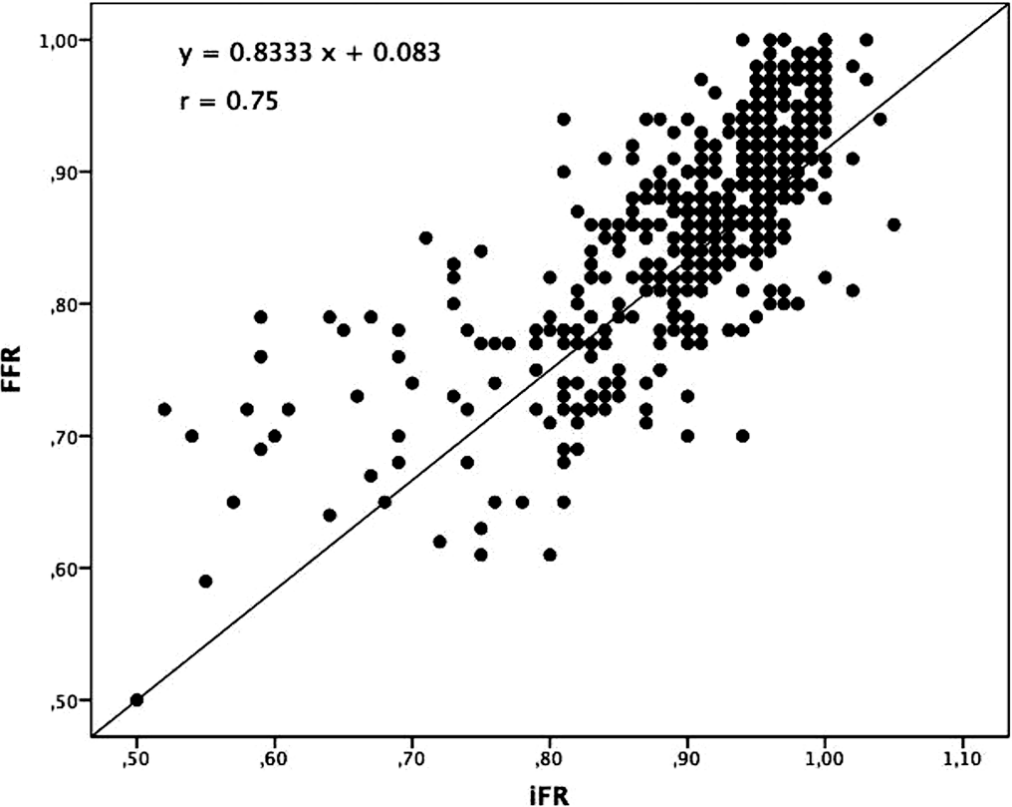
Correlation of instantaneous wave-free ratio and fractional flow reserve

**Fig. 2:**
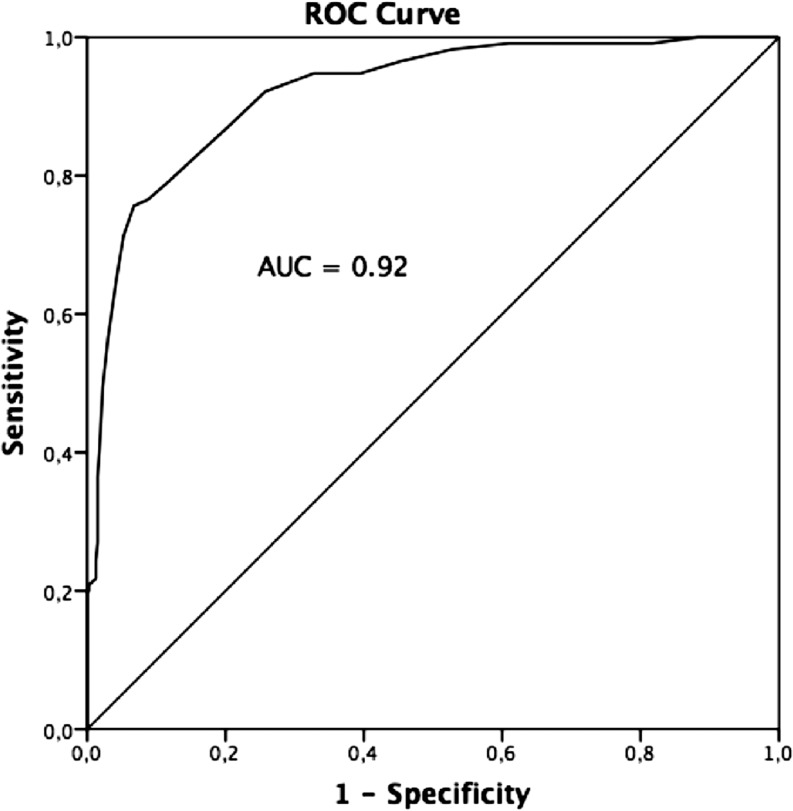
Instantaneous wave-free ratio receiver operating characteristic analysis

Baseline characteristics are summarised in Tab. 1. Mean iFR and FFR were 0.90 ± 0.09 and 0.86 ± 0.08, respectively. iFR correlated well with FFR [*r* = 0.75;* p* < 0.001] (Fig. 1). ROC analysis identified an area under the curve of 0.92 suggesting a high accuracy of iFR as a diagnostic test for FFR (Fig. 2). The estimation of MIC thresholds revealed an iFR of 0.86 (95% confidence interval: 0.90–0.95) as the best cut-off for prediction of an FFR of 0.8 in our population.

**Table 1:** Baseline characteristics

Age, mean ± SD	67 ± 10
Female	110 (31)
Radial approach	258 (73)
Intravenous adenosine	231 (45)
Sinus rhythm	198 (98)
Heart rate	71 (16)
Systolic blood pressure in mm Hg, mean ± SD	146 ± 28
Diastolic blood pressure in mm Hg, mean ± SD	74 ± 15
*Comorbidities*	
– Previous percutaneous coronary intervention	120 (34)
– Previous coronary artery bypass graft	25 (7)
– Prior stroke/transient ischaemic attack	27 (8)
– Hypertension	198 (56)
– Diabetes mellitus	74 (21)
– Hypercholesterolaemia	132 (37)
– Congestive heart failure	28 (8)
– Current smoker	50 (14)
*Coronary artery*	
– Left anterior descending	218 (43)
– Diagonal branch	19 (4)
– Intermediate	14 (3)
– Circumflex	88 (17)
– Obtuse marginal	19 (4)
– Right	108 (21)

*SD* standard deviation

**Fig. 3:**
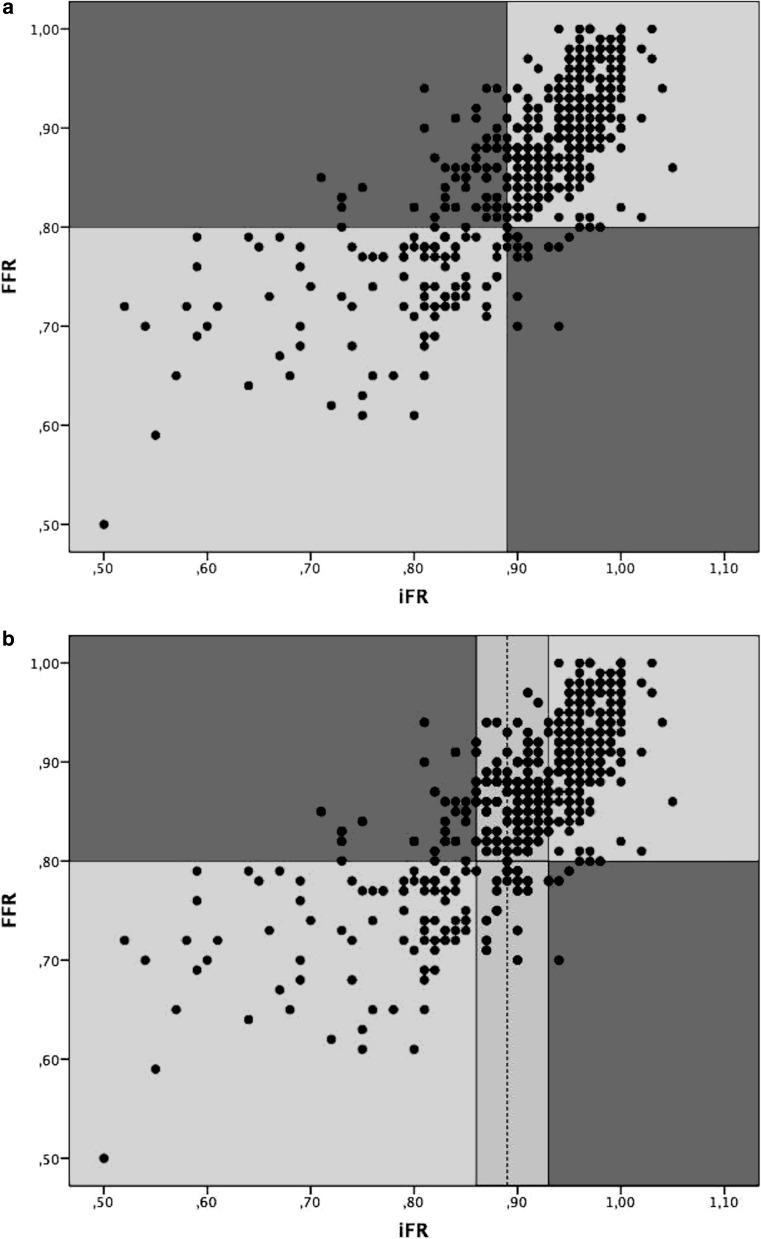
Diagnostic agreement between instantaneous wave-free ratio (iFR) and fractional flow reserve (FFR). **a** iFR-only strategy. **b** Hybrid iFR-FFR approach

The iFR-only strategy using a cut-off of 0.89 showed a diagnostic agreement with the FFR in 420 (82%) lesions (Fig. 3a) with a sensitivity of 87%, a specificity of 80%, a positive predictive value of 56% and a negative predictive value of 96%. Using the hybrid iFR-FFR approach the functional severity of 484 (94%) lesions were accurately assessed (Fig. 3b) with the need of adenosine exposure limited to 178 (35%) lesions.

Of the three false negative results using the hybrid approach two were in males measuring stenoses in the more distal and diffusely diseased left (*n* = 2) and right (*n* = 1) coronary artery (vasospasm during measurement).

Due to diagnostic reclassification using a hybrid iFR-FFR approach over an iFR only approach (Tab. 2) percutaneous coronary intervention was deferred in 372 (72%) stenoses. We observed no statistically significant differences in baseline characteristics between FFR concordant and discordant iFR measurements (data not shown).

**Table 2:** Re-classification using a hybrid iFR-FFR over an iFR-only strategy

Hybrid iFR-FFR strategy	iFR-only strategy				
	True pos.	False pos.	True neg.	False neg.	*Total*
True pos.	100	0	0	11	*111*
False pos.	0	28	0	0	* 28*
True neg.	0	52	320	0	*372*
False neg.	0	0	0	4	* 4*
*Total*	*100*	*80*	*320*	*15*	*515*

*FFR* fractional flow reserve, *iFR* instantaneous wave-free ratio, *neg* negative. *pos* positive

## Discussion

This is the largest, prospective registry of real-time concomitant iFR-FFR measurements to date. These data demonstrate that single iFR measurements are feasible, safe and correlate well with FFR measurements, with a particularly high negative predictive value.

### iFR only

Our objective was to provide additional evidence for the clinical, real-time use of iFR measurements in functional assessment of intermediate coronary artery stenoses. These data are in line with recent observations that iFR has the potential to become such a diagnostic tool [5–7, 12, 13]. All iFR measurements were technically feasible, readily available and a single iFR measurement sufficed. The iFR-only strategy was based on the ‘non-clinically’ derived cut-off of 0.89 and resulted in similar diagnostic agreement with FFR, lower specificity and positive predictive value but higher sensitivity and negative predictive value compared with prior studies [13]. However, following the result of DEFINE-FLAR [6] and iFR-SWEDEHEART [7] the appropriateness of FFR as the gold standard is questionable. Perhaps the clinical trial iMODERN (iFR Guided Multi-vessel revascularizatiOn During percutaneous coronary intervEntion for acute myocaRdial iNfarction; NCT03298659) can provide insight into this matter.

### Hybrid approach

Adopting the previously suggested hybrid approach with an iFR cut-off value for revascularisation (0.86) and deferral (0.93) resulted in an expected substantial improvement in diagnostic agreement. However, when relying upon a hybrid strategy as the solution the outset should be to minimise irreversible actions, in other words, inappropriate revascularisation and its sequelae such as antiplatelet therapy.

### Cut-off values

The diagnostic agreement between FFR and iFR depends on the used cut-off values. Whereas some might argue there is room for debate regarding the optimal FFR cut-off [15, 16], this is particularly true for iFR cut-off values. Although an iFR-guided revascularisation strategy was noninferior to FFR-guided revascularisation in the trials reported by Davies et al. and Götberg et al. [6, 7], outcomes in patients with iFR-guided deferral of revascularisation were not reported. If indeed the clinical outcomes were similar, interventional cardiologists would have more confidence in deferring revascularisation if the iFR is higher than 0.89, and these findings would help to encourage transition to a sole iFR-guided strategy.

Both the iFR-only strategy (0.89) and the ‘iFR grey zone’ (0.86–0.93) are established based on a model, not on clinically derived values [8, 9]. Within our cohort the MIC-derived optimal iFR cut-off value was slightly lower compared with the applied, in other words the accepted, cut-off, opposed to a previous study showing identical values [13]. A recent study by Kobayashi et al. showed that the diagnostic accuracy of iFR depends on the location of the lesion in the coronary tree [17]. In particular, the diagnostic accuracy of iFR was significantly lower than that of FFR for lesions located in the left main or proximal left anterior descending coronary artery; this is probably related to the larger amount of myocardium supplied [17]. This difference may have clinical relevance. Altogether it appears that the hybrid functional assessment of coronary artery stenoses could benefit from more clinical data on optimal cut-off values.

### Discordant measurements

Discordant measurements consisted mainly of false-positive results which is in contrast with prior studies [8, 14]. Further analysis did not reveal a significant difference in patient or haemodynamic characteristics and this observation might be best explained by microvascular dysfunction. We corroborate prior evidence of significantly higher iFR and FFR values in the right coronary and circumflex artery with their branches [data not shown] [13]. Given the vast majority of discordant iFR and FFR measurements consisted of false positive results, these two observations could be linked. How the latter can be explained, physiologically or otherwise, remains as interesting as speculative and requires dedicated further research such as the recently initiated study FiGARO (FFR versus iFR Assessment of Hemodynamic Lesion Significance; NCT03033810).

Additionally, a previous study with direct comparisons between iFR and FFR found also a mismatch in about 20% of cases and they found iFR appeared to correlate better with flow measurements (coronary flow reserve) than FFR [18].

### Clinical implications

iFR measurements eliminate the necessity for adenosine exposure and thereby the associated side effects, additional time and costs as proven by the studies iFR-SWEDEHEART (Evaluation of iFR vs. FFR in Stable Angina or Acute Coronary Syndrome) and DEFINE-FLAIR (Functional Lesion Assessment of Intermediate Stenosis to Guide Revascularisation) [6, 7]. Our data are in line with the evidence. This alone could stimulate more systematic use of functional stenosis assessment. Which is important as it not only answers the question whether or not to revascularise, but also impacts the preferred method of revascularisation [19]. However, the added value of iFR measurements could extend to at least two other important clinical settings. Maybe this is not the case for diffusely diseased coronary arteries or tandem lesions (although FFR is very well suited for single ‘spot’ or non-complex lesions). In such cases a pullback is required to determine the culprit section. Considering that the hyperaemic flow, but not the resting flow, of tandem lesions are interdependent, iFR measurements provide both a more practical and better physiological roadmap of the entire coronary artery [20].

Second, the book on culprit lesion versus complete revascularisation in the setting of ST-elevation myocardial infarction or non-ST-elevation myocardial infarction is still not closed [21]. As such, iFR measurements could potentially provide an adenosine-free practical alternative for which the results of the WAVE trial (Instantaneous Wave-Free Ratio and Fractional Flow Reserve for the Assessment of Non Culprit Lesions in Patients with ST-segment Elevation Myocardial Infarction; NCT02869906) trial are much anticipated. Although iFR may be non-inferior to FFR, as mentioned above, there are some concerns about iFR: first, experimental studies supporting the hypothesis behind iFR are lacking; second, the existence of the wave-free period, upon which iFR is based, has been questioned [22]; and third, outcome studies that compare with FFR have been primarily performed in low-risk patients.

Finally, despite clear non-inferiority (but not independent superiority) of an iFR compared to an FFR guided revascularisation strategy on key clinical endpoints [6, 7] future research to improve current intracoronary diagnostic measurements is necessary to improve our understanding of discrepancies, cut-off values and consequently coronary revascularisation and clinical outcome in high-risk patients [23].

### Limitations

First, as certain technical aspects (e. g. arterial access, route of adenosine administration and subsequent catheter position check) were left at the discretion of the operator this potentially introduced bias, in particular the use of intracoronary nitrates considering its effect beyond the vasotonic constriction of the stenosis. Second, despite the many relevant patient and haemodynamic characteristics that we collected, some important aspects with regard to analysing FFR discordance and concordance could have been omitted. Therefore, based on these data, the possibility of a significant interaction cannot be eliminated. Third, even though these data are from a large all-comer prospective registry, it is not a randomised clinical trial. In other words, all stenoses were assessed by both iFR and FFR and the FFR measurement was leading in the decision to revascularise or defer percutaneous coronary intervention.

## Conclusions

Real-time iFR measurements are easily performed, have a good negative predictive value compared with FFR but the diagnostic accuracy is moderate with 82%. Measurements with iFR have the potential to expose fewer patients to adenosine, reduce procedure time and costs. Further prospective and randomised trials are needed to evaluate specific clinical settings.

## References

[1] White CW, Wright CB, Doty DB (1984). Does visual interpretation of the coronary arteriogram predict the physiologic importance of a coronary stenosis?. N Engl J Med.

[2] Crystal GJ, Klein LW (2015). Fractional flow reserve: physiological basis, advantages and limitations and potential gender differences. Curr Cardiol Rev.

[3] Sen S, Escaned J, Malik IS (2012). Development and validation of an adenosine-independent index of stenosis severity from coronary wave-intensity analysis: results of the ADVISE (ADenosine Vasodilator Independent Stenosis Evaluation) study. J Am Coll Cardiol.

[4] Petraco R, Escaned J, Sen S (2013). Classification performance of instantaneous wave-free ratio (iFR) and fractional flow reserve in a clinical population of intermediate coronary stenoses: results of the ADVISE registry. EuroIntervention.

[5] Petraco R, Park JJ, Sen S (2013). Hybrid iFR–FFR decision-making strategy: implications for enhancing universal adoption of physiology-guided coronary revascularisation. EuroIntervention.

[6] Davies JE, Sen S, Dehbi HM (2017). DEFINE-FLAIR (Functional Lesion Assessment of Intermediate Stenosis to Guide Revascularisation). N Engl J Med.

[7] Götberg M, Christiansen EH, Gudmundsdottir IJ (2017). The Instantaneous Wave-free Ratio versus Fractional Flow Reserve in Patients with Stable Angina Pectoris or Acute Coronary Syndrome (iFR-SWEDEHEART). N Engl J Med.

[8] Petraco R, Al-Lamee R, Gotberg M (2014). Real-time use of instantaneous wave-free ratio: results of the ADVISE in-practice: an international, multicenter evaluation of instantaneous wave-free ratio in clinical practice. Am Heart J.

[9] Johnson NP, Kirkeeide RL, Asrress KN (2013). Does the instantaneous wave-free ratio approximate the fractional flow reserve?. J Am Coll Cardiol.

[10] Finet G, Rioufol G (2012). A new adenosine-independent index of stenosis severity: why would one assess a coronary stenosis differently?. J Am Coll Cardiol.

[11] Pijls NH, Van ’t Veer M, Oldroyd KG (2012). Instantaneous wave-free ratio or fractional flow reserve without hyperemia: novelty or nonsense?. J Am Coll Cardiol.

[12] Rudzinski W, Waller AH, Kaluski E (2012). Instantaneous wave-free ratio and fractional flow reserve: close, but not close enough!. J Am Coll Cardiol.

[13] Härle T, Bojara W, Meyer S (2015). Comparison of instantaneous wave-free ratio (iFR) and fractional flow reserve (FFR)—first real world experience. Int J Cardiol.

[14] Jeremias A, Machara A, Généreux P (2014). Multicenter core laboratory comparison of the instantaneous wave-free ratio and resting Pd/Pa with fractional flow reserve the RESOLVE study. J Am Coll Cardiol.

[15] Pijls NH, van Schaardenburgh P, Manoharan G (2007). Percutaneous coronary intervention of functionally nonsignificant stenosis: 5‑year follow-up of the DEFER study. J Am Coll Cardiol.

[16] Tonino PA, De Bruyne B, Pijls NH (2009). Fractional flow reserve versus angiography for guiding percutaneous coronary intervention. N Engl J Med.

[17] Kobayashi Y, Johnson NP, Berry C (2016). The influence of lesion location on the diagnostic accuracy of adenosine-free coronary pressure wire measurements. JACC Cardiovasc. Interv..

[18] Cook C, Jeremias A, Ahmad Y (2016). Discordance in stenosis classification by pressure-only indices of stenosis severity is related to differences in coronary flow reserve: the RESOLVING DISCORD study, 28th annual Transcatheter Cardiovascular Therapeutics Symposium (TCT). JACC Cardiovasc. Interv..

[19] Montalescot G, Sechtem U, Achenbach S (2013). 2013 ESC guidelines on the management of stable coronary artery disease. Eur Heart J.

[20] Nijer SS, Sen S, Escaned JJ (2014). Pre-angioplasty instantaneous wave-free ratio pullback provides virtual intervention and predicts hemodynamic outcome for serial lesions and difuse coronary artery disease. JACC Cardiovasc. Interv..

[21] Smits PC, Abdel-Wahab M, Neumann FJ (2017). Fractional flow reserve–guided multivessel angioplasty in myocardial infarction. N Engl J Med.

[22] Van ’t Veer M, Pijls NHJ, Hennigan B (2017). Comparison of different diastolic resting indexes to IFR. J. Am. Coll. Cardiol..

[23] Piek JJ (2017). Coronary physiology revisited. Neth Heart J.

